# Application analysis of two nucleic acid detection systems in blood detection of hepatitis B virus, hepatitis C virus and human immunodeficiency virus

**DOI:** 10.7717/peerj.20365

**Published:** 2025-11-27

**Authors:** Tao Wang, Jianwei Zhang

**Affiliations:** Changzhou Blood Center, Changzhou, China

**Keywords:** Nucleic acid testing, Blood screening, Hepatitis B virus, Hepatitis C virus, Human immunodeficiency virus, Diagnostic efficiency

## Abstract

**Objective:**

This study aimed to compare the diagnostic efficacy between the Kehua and Roche nucleic acid testing (NAT) systems for detecting hepatitis B virus (HBV), hepatitis C virus (HCV), and human immunodeficiency virus (HIV) in blood donation screening.

**Methods:**

We analyzed retrospective data from 670,775 transient blood donation samples collected between 2016 and 2024. Key performance indicators (KPIs) included the types of reactive samples, effective split rate, effective reaction rate, single pool invalid number, batch invalid number, and pooling missing samples. Annual trends in cycle threshold (CT) value distributions and overall system performance were also evaluated.

**Results:**

Among 419 reactive samples (Kehua: 202; Roche: 217), no significant differences were observed in the effective split rates (*P* > 0.05) or overall specimen reaction rates (*P* > 0.05) between the two systems. The HCV detection rate was significantly lower for the Kehua system (*P* < 0.05), while the detection rates for HBV and HIV showed no significant inter-system differences (*P* > 0.05). The separation efficiency of reactive pools varied significantly across different CT value ranges (*P* < 0.05). The Kehua system demonstrated stable annual effective split and reaction rates (*P* > 0.05), whereas the Roche system exhibited significant annual fluctuations in these metrics (*P* < 0.05). The Kehua system had significantly fewer single pool invalid numbers and pooling omissions than the Roche system (*P* < 0.05).

**Conclusion:**

The Kehua and Roche NAT systems demonstrated comparable overall performance in blood screening, with Kehua proving non-inferior to Roche. Kehua’s advantages included fewer invalid tests and fewer pooling errors, which could reduce economic and time costs. The Roche system exhibited higher automation, supporting continuous batch processing. The observed CT value-dependent separation efficiency suggests potential for protocol optimization in detecting low viral load samples.

## Introduction

Hepatitis B virus (HBV), hepatitis C virus (HCV), and human immunodeficiency virus (HIV) are major blood-borne pathogens that pose a significant threat to transfusion safety worldwide (Al-Amad, 2024; Laperche et al., 2023; Mitterreiter et al., 2022). To mitigate this risk, blood banks globally implement rigorous screening of donated blood for these infectious agents to prevent transfusion-transmitted infections (Coste et al., 2005). For decades, serological immunoassays have been the traditional method for detecting viral antibodies or antigens. However, these assays have inherent limitations, including the inability to detect infections during the immunologically silent window period, issues with viral variants, and occult infections (Sang et al., 2021; Paul, Ostermann & Wei, 2020; Zhao et al., 2015). Furthermore, the clinical management and diagnosis of patients co-infected with multiple blood-borne viruses present additional complexities, underscoring the critical need for highly sensitive and specific screening methods (Shahriar et al., 2022).

The adoption of nucleic acid amplification testing (NAT) marked a revolutionary advancement in blood safety. Initially implemented in the United States in 1999 under a research protocol approved by the US Food and Drug Administration (FDA), NAT was introduced to directly detect HIV-1 and HCV Ribonucleic Acid (RNA), effectively shortening the window period and enhancing the detection of occult infections (Stramer et al., 2004; Busch et al., 2000). This technology has since become a cornerstone of blood safety protocols globally.

In China, two predominant NAT platforms employed are the Kehua and Roche nucleic acid blood screening systems. While both systems are widely used, a comprehensive comparative analysis of their operational efficiency and performance in real-world, high-throughput settings is needed. This study aimed to retrospectively analyze the screening results of voluntary, unpaid blood donors in our region from January 2016 to December 2024. We specifically compared the performance of the Kehua and Roche systems by evaluating key metrics, including the effective split rate, effective reaction rate, single pool invalid number, batch invalid number, and pooling missing samples. The objective of this comparison was to elucidate the respective advantages and limitations of each system, providing data-driven insights to optimize blood screening strategies.

## Materials and Methods

### Study subjects

The study utilized residual serum samples from voluntary, unpaid blood donors in the region, collected between January 2016 and December 2024. A total of 670,775 samples that were non-reactive (negative) in the enzyme-linked immunosorbent assay (ELISA) screening were subsequently included for nucleic acid testing (NAT). The study protocol was reviewed and approved by the Institutional Review Board (IRB) of Changzhou Blood Center. All blood donors had provided written informed consent prior to donation, which explicitly permitted the use of their residual samples for transfusion-related research, including nucleic acid testing methodology studies. This study was conducted under the provisions of that consent. All donor identities were permanently delinked from the test data prior to analysis, ensuring confidentiality. All procedures were performed in accordance with the ethical standards of the Declaration of Helsinki.

**Figure 1:**
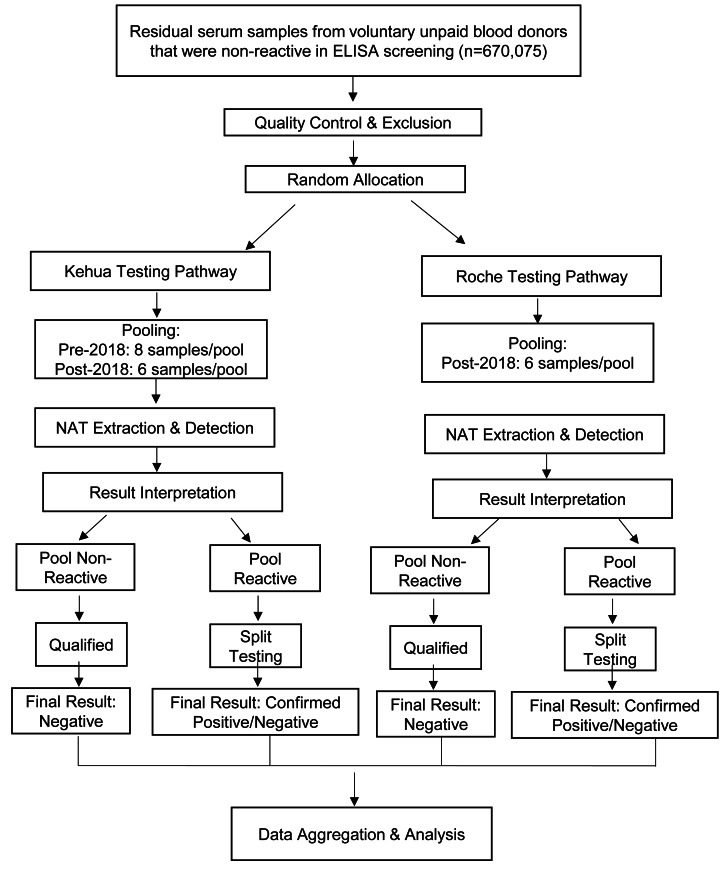
Flowchart of the parallel comparison process between the Kehua and Roche NAT systems. This study employed a parallel-testing design. ELISA-non-reactive samples, after quality control (*e.g.*, exclusion of hemolyzed/lipemic samples), were randomly allocated to either the Kehua or Roche system for independent NAT processing. Subsequent data analysis was based on aggregated performance metrics derived from two large, independent cohorts.

Sample Inclusion and Exclusion Criteria: All samples had tested non-reactive for hepatitis B surface antigen (HBsAg), anti-HCV, and anti-HIV using standard ELISA screening. In accordance with laboratory quality control protocols, pre-analytical processing was applied to exclude samples with potential interference in test results. Specifically, samples exhibiting gross hemolysis (evidenced by reddish discoloration) or significant lipemia (indicated by turbid plasma) were excluded. Additionally, samples with insufficient volume or improper collection procedures (*e.g.*, clotting issues) were not included in the analysis. These measures ensured that all enrolled samples met the required quality standards, thereby minimizing the risk of false-negative results or technical interference. The overall testing and comparison workflow for the included samples is visually summarized in Fig. 1.

### Reagents and instruments

#### ELISA kit was used for the serological test

The initial test included HBsAg diagnostic kit (Shanghai Kehua Bioengineering Co., Ltd., Shanghai, China), HCVAb diagnostic kit (Beijing Wantai biopharmaceutical Co., Ltd., Beijing, China), Anti-HIV diagnostic kit (Zhuhai Lizhu Reagent Co., Ltd., Zhuhai, China; Beijing Wantai Biological Pharmaceutical Co., Ltd.). The retest included HBsAg diagnostic kit, HCVAb diagnostic kit and anti-HIV diagnostic kit (all belong to Yingke Xinchuang (Xiamen) Technology Co., Ltd., China).

Nucleic acid detection used hepatitis B virus, hepatitis C virus, and human immunodeficiency virus (type 1) nucleic acid detection kit (PCR fluorescence method) (Shanghai Kehua Bioengineering Co., Ltd.) and hepatitis B virus, hepatitis C virus, human immunodeficiency virus (type 1 + 2) nucleic acid detection kit (PCR fluorescence method) cobas TaqScreen MPX Test, Version 2.0 (Roche Molecular Systems, Inc, Pleasanton, CA, USA), The internal quality control samples were purchased from Beijing Kangchestan company. All above reagents passed batch inspection and are used within the validity period.

#### Kehua nucleic acid blood screening system

The Kehua NAT system (Kehua Bio-engineering, China) is an integrated automated platform that executes the complete NAT workflow through coordinated operation of its extraction (Microlab STAR IVD; Hamilton Microlab, Hamilton Bonaduz AG, Bonaduz, Switzerland) and amplification (7500 Real-Time PCR System; Thermo Fisher Scientific, Waltham, MA, USA) modules. This system performs automated sample pipetting, magnetic bead-based nucleic acid extraction and purification, PCR amplification, and real-time fluorescent detection in a continuous processing sequence.

#### Roche nucleic acid blood screening system

The Roche cobas^®^ S201 system (Roche Diagnostics, Basel, Switzerland) is a fully integrated, automated platform that performs all steps of the NAT workflow, including sample preparation (pipetting, nucleic acid extraction, and purification), amplification (PCR), and real-time fluorescent detection in a single, continuous batch process.

### Nucleic acid detection procedure

Both detection systems employed a mixed-sample testing mode for the simultaneous detection of HBV DNA, HCV RNA, and HIV RNA. The Kehua system utilized an 8-sample pooling mode before November 29, 2018, and switched to a 6-sample pooling mode thereafter. The Roche system consistently employed a six-sample pooling mode. Samples from non-reactive pools were deemed qualified. Reactive pools underwent reflex split testing, with the split result serving as the final determination. Each testing batch included negative controls, positive controls, and internal quality control samples to monitor assay performance.

### Key performance indicators

The study evaluated the following quality metrics for nucleic acid testing systems:

#### Effective split rate

*Definition*: The proportion of initially reactive (positive) pooled samples that maintain reactivity when split into individual tests.

*Calculation*: Effective Split Rate = Number of confirmed reactive individual tests/Number of reactive pools ×100%.

#### Single pool invalid number

*Definition*: The count of invalid pools (either mixed or individual tests) due to technical failures within a batch. This metric reflects system stability and indirectly indicates cost-effectiveness by measuring re-testing requirements.

Criteria for designating an invalid pool: The criteria for designating an invalid pool in this study strictly adhered to the standard operating procedures provided by the respective reagent manufacturers. For the Roche system, as per the cobas MPX assay instructions, a pool (either mixed or individual) was automatically flagged as “Invalid” by the software if all viral targets (HBV, HCV, HIV) were undetected and the Internal Control (IC) failed simultaneously, necessitating re-extraction and re-testing. The fundamental principle was that any sample or pool with a failed IC amplification was deemed invalid. This criterion ensures the robust identification and exclusion of any potential amplification failures arising from factors such as low extraction efficiency, the presence of PCR inhibitors, or reaction setup errors. It is important to note that samples with very high Ct values (*e.g.*, approaching 40) were still considered valid positive results and reported as long as the IC amplification was successful.

#### Effective reaction rate

*Definition*: The proportion of blood samples showing nucleic acid test (NAT) reactivity.

*Calculation*: Effective Reaction Rate = Number of NAT −reactive samples (RS)/Total tested samples ×100%.

#### Batch invalid number

Definition: The number of entire testing batches invalidated due to systemic failures (*e.g.*, equipment malfunction, contamination). Used with Single Pool Invalid Number to directly assess system stability and economic impact.

#### Pooling missing samples

Definition: The count of target samples inadvertently omitted during automated pooling, reflecting the reliability of the mixing process.

### System automation and operational differences

Notable differences exist between the Kehua and Roche systems in terms of automation integration and manual operation requirements:

The Roche cobas^®^ S201 system is a fully integrated and continuous automated platform. It automates all steps—including sample preparation (pipetting, nucleic acid extraction, and purification), PCR amplification, and real-time fluorescent detection. Operators need only load samples and reagents initially; the system then processes a batch (maximum of 24 tubes, equivalent to 120 samples) autonomously in approximately 5.5 h with minimal manual intervention, ensuring process continuity and result consistency.

The Kehua NAT system is a modular integrated system where the nucleic acid extraction module and the amplification/detection module operate in coordination. Its workflow requires more manual involvement, including, but not limited to: manual preparation of lysis buffer and internal control mix, manual preparation of HBV/HCV/HIV amplification reaction mixtures, and manual transfer of sample plates between modules. Processing a maximum batch (32 tubes, equivalent to 180 samples) requires approximately 5 h, with technicians needed for multiple operational steps.

In summary, while the total hands-on time for a single batch is similar (∼5 h), the Roche system offers a higher degree of automation and operational continuity, whereas the Kehua system provides flexibility at the cost of requiring more manual technical steps.

### Statistical analysis

The data in this study were statistically analyzed using SPSS 26.0 software (version 26.0; IBM Corp., Armonk, NY, USA). Categorical data were expressed as number of cases and percentage, and the independent sample chi-square test was used. A *P* < 0.05 showed that it was statistically significant.

## Results

### Effective split rate and sample reaction rate of the two systems

From January 1, 2016, to December 31, 2024, a total of 670,775 samples were tested by Kehua nucleic acid blood screening system and Roche nucleic acid blood screening system, including 347,033 cases tested by Kehua nucleic acid blood screening system and 323,742 cases tested by Roche nucleic acid blood screening system. The effective split rate of the Kehua nucleic acid blood screening system was 53.37%, and that of the Roche nucleic acid blood screening system was 51.67%. There was no significant difference between Kehua nucleic acid blood screening system and Roche nucleic acid blood screening system (*P* > 0.05). The effective reaction rate of the Kehua nucleic acid blood screening system was 0.058%, and that of the Roche nucleic acid blood screening system was 0.067%. There was no significant difference between Kehua nucleic acid blood screening system and Roche nucleic acid blood screening system (*P* > 0.05). The number of RS was greater than the number of split-reactive pools because one pool could separate multiple positive samples. The results are shown in Table 1.

**Table 1:** Effective split rate and sample reaction rate of the two systems.

Systems	Detection quantity (cases)	Total number of pools (cases)	Number of mixed reactive pools (cases)	Number of split-reactive pools (cases)	Effective split rate (%)	Number of reactive samples (cases)	Effective reaction rate (%)
Kehua	347,033	54,389	371	198	53.37	202	0.058
Roche	323,742	54,839	418	216	51.67	217	0.067
*χ* ^2^	NA	NA	NA	0.226		2.088	
*P*-value	NA	NA	NA	0.634		0.149	

**Notes.**

The effective split rates and effective reaction rates between the Kehua and Roche systems showed no statistically significant differences (*P* > 0.05).

### Categories of RS detected by the two systems

From January 1, 2016, to December 31, 2024, a total of 419 RS were detected by the Kehua and Roche nucleic acid blood screening systems. Among the RS detected by the Kehua blood screening system, 199 were HBV DNA RS, accounting for 98.51% of the total RS detected by the Kehua system, two were HCV RNA RS (0.99%), and one was HIV RNA RS (0.50%). In the RS detected by the Roche nucleic acid blood screening system, 205 were HBV DNA RS, accounting for 94.47% of the total RS detected by the Roche system, nine were HCV RNA RS (4.15%), and three were HIV RNA RS (1.38%). The differences in the detection rates of HBV and HIV infections between the two systems were not statistically significant (*P* > 0.05). However, the detection rate of HCV by Kehua was significantly lower than that by Roche (*P* < 0.05). The categories of RS detected by the two systems are shown in Table 2.

### Distribution of CT values for reactive pools and split reactive pools in HBV mixed samples

In the Kehua system: The highest number of reactive pools in mixed testing was observed in the range of 33 ≤ CT < 35, accounting for 37.50% of the total. The lowest number of reactive pools in mixed testing was observed when CT ≥ 40, accounting for 5.71%. The highest effective separation rate was observed when CT < 33, while the lowest effective separation rate was observed when CT ≥ 40. The differences in the number of reactive pools across different CT value ranges were statistically significant (*P* < 0.001). The highest number of split reactive pools was observed in the range of 33 ≤ CT <35, accounting for 38.97%. The differences in the number of split reactive pools across different CT value ranges were statistically significant (*P* < 0.001). In the Roche system: The highest number of reactive pools in mixed testing was observed in the range of 37≤CT< 40, accounting for 49.51%. The lowest number of reactive pools in mixed testing was observed when CT < 33, accounting for 2.96%. The highest effective separation rate was observed in the range of 33 ≤ CT < 35, while the lowest effective separation rate was observed when CT ≥ 40. The differences in the number of reactive pools across different CT value ranges were statistically significant (*P* < 0.001). The highest number of split reactive pools was observed in the range of 37 ≤ CT < 40, accounting for 52.20%. The lowest number of split reactive pools was observed when CT ≥ 40, accounting for 4.391%. The differences in the number of split reactive pools across different CT value ranges were statistically significant (*P* < 0.001). The distribution of CT values for reactive pools and split reactive pools in HBV mixed samples is shown in Table 3.

**Table 2:** Types of reactive specimens detected by two systems. The detection rate for HCV was significantly lower in the Kehua system compared to the Roche system (*P* = 0.026), while no significant differences were found for HBV and HIV (*P* > 0.05).

Systems	HBV detection rate (‱)	HCV detection rate	HIV detection rate
Kehua	5.73	0.06	0.05
Roche	6.33	0.28	0.09
*χ* ^2^	0.995	4.960	1.145
*P*-value	0.319	0.026	0.285

**Table 3:** Distribution of CT Values for reactive pools and split reactive pools in HBV mixed samples. The distribution of reactive pools and split-reactive pools across CT value ranges differed significantly between the two systems (*P* < 0.001), with Kehua showing the highest number of reactive pools at 33 ≤ CT<35 and Roche at 37 ≤ CT<40.

CT value	Number of reactive pools in mixed testing		Proportion of reactive pools in mixed testing (%)		Number of split reactive pools		Split reactive rate (%)		Effective separation rate (%)	
	Kehua	Roche	Kehua	Roche	Kehua	Roche	Kehua	Roche	Kehua	Roche
CT<33	92	12	25.00	2.96	68	11	34.87	5.37	73.91	91.67
33≤CT<35	138	14	37.50	3.45	76	13	38.97	6.34	55.07	92.86
35≤CT<37	70	100	19.02	24.63	35	65	17.95	31.71	50.00	65.00
37≤CT<40	47	201	12.77	49.51	12	107	6.15	52.20	25.53	53.23
40≤CT	21	79	5.71	19.46	4	9	2.05	4.39	19.05	11.39
*χ* ^2^	135.414	369.686	NA	NA	133.974	232.927	NA	NA	NA	NA
*P*-value	<0.001	<0.001	NA	NA	<0.001	<0.001	NA	NA	NA	NA

### Distribution of CT values for split RS in HBV testing

In the Kehua system: The highest number of split RS was observed when CT < 33, accounting for 59.30% of the total. The lowest number of split RS was observed when CT ≥ 40, accounting for 0.50%. The differences in the number of split RS across different CT value ranges were statistically significant (*P* < 0.001). In the Roche system: The highest number of split RS was observed in the range of 35 ≤ CT < 37, accounting for 33.17% of the total. The lowest number of split RS was observed when CT ≥ 40, accounting for 3.41%. The differences in the number of split RS across different CT value ranges were statistically significant (*P* < 0.001). The distribution of CT values for split RS in HBV testing is shown in Table 4.

**Table 4:** Distribution of CT values for split reactive specimens in HBV testing. The distribution of split-reactive specimens across CT value ranges was significantly different between the Kehua and Roche systems (*P* < 0.001). Most reactive specimens in the Kehua system had CT < 33, whereas in the Roche system they were predominantly in the 35  ≤ CT  <  37 range.

CT value	Number of split reactive specimens		Proportion of split reactive specimens (%)	
	Kehua	Roche	Kehua	Roche
CT<33	118	30	59.30	14.63
33≤CT<35	50	41	25.13	20.00
35≤CT<37	24	68	12.06	33.17
37≤CT<40	6	59	3.02	28.78
40≤CT	1	7	0.50	3.41
*χ* ^2^	286.332	71.037	NA	NA
*P*-value	<0.001	<0.001	NA	NA

### Annual nucleic acid detection of Kehua nucleic acid blood screening system

From January 1, 2016, to December 31, 2024, the annual nucleic acid detection of the Kehua nucleic acid blood screening system was shown in Table 5. On November 29, 2018, Kehua nucleic acid system changed from eight mixed samples/per pool to six mixed samples/per pool. There was no significant difference in the effective split rate of the Kehua nucleic acid blood screening system each year (*P* > 0.05). There was no significant difference in the effective reaction rate of samples in each year of the Kehua nucleic acid blood screening system (*P* > 0.05).

**Table 5:** Annual nucleic acid detection of Kehua nucleic acid blood screening system. No significant annual differences were observed in the effective split rate or effective reaction rate for the Kehua system (*P* > 0.05), despite a change in the pooling strategy from 8 to 6 samples per pool in November 2018.

Year	Detection quantity (cases)	Total number of pools (cases)	Number of mixed test reaction pools (cases)	Number of split-reactive pools (cases)	Effective split rate (%)	Number of reactive samples (cases)	Effective reaction rate (%)
2016	45,218	5,753	70	33	47.14	33	0.073
2017	23,385	3,060	16	9	56.25	9	0.038
2018	30,320	4,019	28	22	78.57	23	0.076
2019	28,473	4,774	26	16	61.54	18	0.063
2020	32,916	5,506	31	17	54.84	17	0.052
2021	44,633	7,471	52	26	50.00	26	0.058
2022	50,328	8,428	53	26	49.06	26	0.052
2023	44,368	7,416	47	21	44.68	22	0.050
2024	47,392	7,962	48	28	58.33	28	0.059
*χ* ^2^	NA	NA	NA	11.57		6.20	
*P*-value	NA	NA	NA	0.171		0.625	

### Annual nucleic acid detection of Roche nucleic acid blood screening system

From January 1, 2016, to December 31, 2024, the annual nucleic acid testing performance of the Roche nucleic acid blood screening system is shown in Table 6. There were no statistically significant differences in the annual effective split rate of the Roche nucleic acid blood screening system (*p* > 0.05). However, there were statistically significant differences in the annual specimen reaction rates of the Roche nucleic acid blood screening system (*p* = 0.002).

**Table 6:** Annual nucleic acid detection of Roche nucleic acid blood screening system. No significant annual differences were found in the effective split rate for the Roche system (*P* > 0.05), but the effective reaction rate showed significant variation across years (*P* = 0.002).

Year	Detection quantity (cases)	Total number of pools (cases)	Number of mixed test reaction pools (cases)	Number of split-reactive pools (cases)	Effective split rate (%)	Number of reactive samples (cases)	Effective reaction rate (%)
2016	15,614	2,672	20	13	65.00	13	0.083
2017	41,853	7,070	73	48	65.75	48	0.115
2018	39,359	6,625	51	29	56.86	29	0.074
2019	46,765	7,904	66	32	48.48	32	0.068
2020	44,357	7,503	67	30	44.78	30	0.068
2021	37,476	6,365	38	14	36.84	14	0.037
2022	35,868	6,091	36	15	41.67	15	0.042
2023	36,401	6,171	33	19	57.58	20	0.055
2024	26,049	4,438	34	16	47.06	16	0.061
*χ* ^2^	NA	NA	NA	14.84		24.38	
*P*-value	NA	NA	NA	0.062		0.002	

### Single pool invalid number, batch invalid number and spooling missing samples of the two systems

Single pool invalid number in the Kehua system was significantly lower than that in the Roche system (*P* < 0.001). The number of pooling missing samples in the Kehua system was also significantly lower than that in the Roche system (*P* < 0.001). The details are shown in Table 7.

## Discussion

The proportion of HBV RS in the two systems is higher than that of HIV RS and HCV RS. This trend is consistent with previous research results. However, previous studies have attributed the possible reasons why the detection rate of HIV and HCV is lower than that of HBV to the easy degradation of samples, assay sensitivity of the detection system and ELISA screening (Sun et al., 2021). While these are valid technical considerations, the fundamental driver of this observed disparity is the underlying epidemiological prevalence: the infection rates of HIV and HCV in the general population are significantly lower than that of HBV. Relevant literature shows that around the world, there are about 350–400 million patients infected with HBV, 33.2 million patients infected with HIV and 160 million patients infected with HCV (Davlidova et al., 2021). In China, the number of people infected with HBV is about 140 million (Wang et al., 2019), the number of people infected with HIV is about 1.05 million (Zhang et al., 2021), and the number of people infected with HCV is about 7.6 million (Qu et al., 2021). There are great differences in the number of patients infected with the three viruses, which is the main reason for the difference in the proportion of the detection of the three viruses.

**Table 7:** Comparison of invalid single pools, batch invalid number, and pooling missing samples between the two systems. The Kehua system had a significantly lower number of invalid single pools and pooling missing samples compared to the Roche system (*P* < 0.001). No significant difference was found in the number of invalidated batches (*P* > 0.05).

Systems	Single pool invalid number	Batch invalid number	Pooling missing samples
Kehua	348	30	9
Roche	729	44	92
*χ* ^2^	132.975	0.034	74.162
*P*-value	<0.001	0.853	<0.001

The effective split rate of the Kehua nucleic acid blood screening system is 53.37%. This data result is within the range of previous research results in other regions, with an approximate range of 34.48% to 65.71% (Sun et al., 2021). The effective reaction rate of the Kehua nucleic acid blood screening system is 0.058%, which is within the range of previous research results in other regions, and the range of effective reaction rate in other regions is roughly 0.030% to 0.083 (Sun et al., 2021). The effective split rate of the Roche nucleic acid blood screening system is 51.67%, which is slightly lower than that of other regions, including 56.25% in Jinan (Sun et al., 2021), and 52.22% in Baoji (Wang et al., 2018) and 62.31% in Zhengzhou (Li et al., 2015). The effective reaction rate of the Roche nucleic acid blood screening system is 0.067%, which is within the numerical range of other regions. The numerical differences in the effective split and reaction rates observed across regions for both systems could be influenced by local donor prevalence, sample handling protocols, or other factors. A future meta-analysis would be required to determine if these differences are statistically significant and to identify their root causes. The number of RS is greater than the number of split-reactive pools because one pool can separate multiple positive samples. There was no significant difference in the effective split rate and effective reaction rate between the two systems. It is consistent with the research results of Jinan, Baoji and other places. However, some studies have pointed out that the Roche nucleic acid blood screening system has higher sensitivity, effective split rate, and effective reaction rate than the Kehua nucleic acid blood screening system and has statistical significance. The incidence of HBV, HCV and HIV in blood donors was relatively constant. The two systems randomly detected blood donor samples, and the analysis of the two systems in the blood donor population detection rates of HBV, HCV and HIV showed no significant difference. It shows that the two systems can play the same role in ensuring the safety of blood transfusion under the existing detection technology.

This study found that the HCV detection rate of Kehua is lower than that of Roche. However, further validation with an expanded dataset is still required. Furthermore, the observed disparity in HCV detection rates, with the Kehua system showing lower sensitivity, is likely multifactorial. This can be attributed to the inherent analytical sensitivity difference between the assays, the greater susceptibility of HCV RNA to degradation compared to HBV DNA during storage and handling, and the potential for primer/probe mismatches due to the high genetic variability of HCV. Therefore, the lower detection rate is more indicative of the specific reagent’s performance characteristics than a fundamental limitation of the platform itself, underscoring the importance of assay sensitivity in NAT system comparisons. Due to the small number of detected cases of HCV and HIV, only the CT value distribution of HBV was statistically analyzed in this study. The results in Table 3 showed that in the Kehua system, the CT range of 33 ≤ CT < 35 had the highest number of reactive pools. In contrast, for the Roche system, the highest number of reactive pools was observed in the 37 ≤ CT < 40 range, and the differences between these CT value groups were statistically significant. This result indicated that the CT value distribution of the reactive pools is different between the two systems. The results in Table 4 showed that, in the Kehua system, when CT < 33, the number of split-reacting specimens was the largest. When CT ≥ 40, the number of split-RS was the least. And with the increase of CT value, the number of RS in each group decreased. In the Roche system, when 35 ≤ CT < 37, the number of RS was the largest. When CT ≥ 40, the number of split responding specimens was the least, and the difference between groups was statistically significant. The result indicated that the distribution of CT values of the two systems for splitting reactive pools was also different. This may be related to different system performance. The results in Table 3 showed that although the distribution of CT values of reactive pools in the mixed detection of the two systems was different, the lower the CT value of the mixed samples, the higher the effective split rate, which was consistent with the results of previous studies (Sun et al., 2021). Notably, the difference in the optimal CT value range between the two systems (Kehua: 33–35; Roche: 37–40) bears clinical relevance, as Roche’s detection capability at higher CT values (*i.e.,* lower viral loads) suggests a potential advantage in identifying window-period infections and reducing the residual risk of transfusion transmission.

On November 29, 2018, Kehua nucleic acid system changed from eight mixed samples/per pool to six mixed samples/per pool. However, there was no statistical difference between the effective split rate and effective reaction rate of the Kehua nucleic acid blood screening system from 2016 to 2025. The results indicate that the upgrade in Kehua did not significantly impact the effective split rate and effective reaction rate. There was a significant difference between the effective split rate and effective reaction rate in the annual Roche nucleic acid blood screening system. The differences in specimen reaction rates across different years for the Roche nucleic acid blood screening system were statistically significant.

Unlike other studies, this research also compared single pool invalid number, batch invalid number, and pooling missing samples between the two systems. Single pool invalid number and batch invalid number of Kehua is less than that of Roche. The main reason for batch invalid of the Roche system is hardware failure and the invalid internal control, and the main reason for the invalidation of a single pool is that the amplification curve is not standard, and the system judges invalid. The main reason for the invalid sorties of the Kehua system is the invalid amplification of the internal control and the internal reference, and the main reason for the invalidity of the single pool is the invalid amplification of the internal reference. Compared with the Roche nucleic acid blood screening system, the Kehua nucleic acid blood screening system has better time and economic benefits. It may be related to the high automation of the Roche nucleic acid blood screening system. The system can fully automate the collection of blood samples, nucleic acid extraction, amplification and result analysis and judgment. A high degree of automation can significantly reduce human intervention. However, once the instrument fails, it needs to be tested again during testing. To some extent, it increases the cost and time of detection (Li, Su & Zheng, 2021). The reagents of Kehua system need to be manually configured, and factors such as personnel operation and reagent stability may result in invalid results. Therefore, strengthening personnel training and upgrading system automation is an effective means to improve the detection efficiency of Kehua system.

In conclusion, both systems have their own advantages in blood testing. The detection performance of the Kehua nucleic acid blood screening system is not inferior to that of the Roche nucleic acid blood screening system, and it can screen unqualified blood from the negative samples of the enzyme immunoassay screening, which more effectively guarantees the safety of blood transfusion. Single pool invalid number and pooling missing samples in the Kehua system was lower than that in the Roche system, which to some extent reduced both time and economic costs. The Roche system is highly automated, capable of continuous batch detection, and has the advantage of detection efficiency in the case of a large number of samples with the method of detecting nucleic acid after enzyme immunoassay.

##  Supplemental Information

10.7717/peerj.20365/supp-1 Supplemental Information 1 Dataset

## Additional Information and Declarations

### Data Availability

The following information was supplied regarding data availability: The raw data is available in the Supplemental File.
